# Identification of Crocetin as a Dual Agonist of GPR40 and GPR120 Responsible for the Antidiabetic Effect of Saffron

**DOI:** 10.3390/nu15224774

**Published:** 2023-11-13

**Authors:** Xiaodi Zhao, Dohee Ahn, Gibeom Nam, Jihee Kwon, Songyi Song, Min Ji Kang, Hyejin Ahn, Sang J. Chung

**Affiliations:** 1 Department of Biopharmaceutical Convergence, Sungkyunkwan University, Suwon 16419, Republic of Korea; zhaoxiaodi1019@gmail.com (X.Z.); skarlqja12@g.skku.edu (G.N.); yg1549@naver.com (J.K.); songe1997@naver.com (S.S.); minjii_q@naver.com (M.J.K.); hyejin0930@skku.edu (H.A.); 2 School of Pharmacy, Sungkyunkwan University, Suwon 16419, Republic of Korea; ehgml94@naver.com

**Keywords:** crocetin, GPR40/120 dual agonist, glucose-stimulated insulin secretion (GSIS), glucagon-like peptide (GLP)-1

## Abstract

Crocin, a glycoside of crocetin, has been known as the principal component responsible for saffron’s antidiabetic, anticancer, and anti-inflammatory effects. Crocetin, originating from the hydrolytic cleavage of crocin in biological systems, was subjected to ligand-based virtual screening in this investigation. Subsequent biochemical analysis unveiled crocetin, not crocin, as a novel dual GPR40 and GPR120 agonist, demonstrating a marked preference for GPR40 and GPR120 over peroxisome proliferator-activated receptors (PPAR)γ. This compound notably enhanced insulin and GLP-1 secretion from pancreatic β-cells and intestinal neuroendocrine cells, respectively, presenting a dual mechanism of action in glucose-lowering effects. Docking simulations showed that crocetin emulates the binding characteristics of natural ligands through hydrogen bonds and hydrophobic interactions, whereas crocin’s hindered fit within the binding pocket is attributed to steric constraints. Collectively, for the first time, this study unveils crocetin as the true active component of saffron, functioning as a GPR40/120 agonist with potential implications in antidiabetic interventions.

## 1. Introduction

Diabetes mellitus is one of the leading causes of morbidity and mortality globally, and the burden of this disease has not been adequately addressed by the development of therapeutics. Type 2 diabetes mellitus (T2DM) is a chronic metabolic disorder characterized by hyperglycemia. It is caused by insulin resistance, which is impaired insulin action in target tissues, and insufficient secretion of insulin from pancreatic β-cells [1]. The pro-longed synergy of hyperglycemia with other metabolic abnormalities in patients with diabetes can lead to organ damage and, ultimately, disabling and life-threatening consequences [2]. In the practical approach to managing patients, biguanides, sulfonylureas, and thiazolidinediones (TZDs) are usually utilized with the risks of heart failure, hypo-glycemia, renal impairment, and weight gain [3]. Consequently, research institutions and pharmaceutical companies throughout the world have worked on developing new, and safer anti-T2DM targets to produce better-tolerated antidiabetic medications.

Apart from being vital sources of energy, free fatty acids (FAs) also function as signaling molecules that control insulin secretion and sensitivity, inflammation, body weight, and various other metabolic processes [1]. Especially, the beneficial effects of omega-3 fatty acids (ω-3 FAs) have been thoroughly documented due to their physiological function in stimulating insulin and gut hormone secretion, improving insulin sensitivity, anti-inflammatory effects, increasing glucose uptake, preventing metabolic disorder, and enabling homeostasis of healthy fat tissue [2]. The medium- to long-chain free fatty acids, including linoleic acid (LA), eicosatrienoic acid, and docosahexaenoic acid (DHA), are known as endogenous ligands of G-protein-coupled receptor 40 (GPR40) and G-protein-coupled receptor 120 (GPR120). GPR40, also known as free fatty acid receptor 1 (FFA1), is a GPR that is primarily expressed in β-cells of the pancreas and enteroendocrine L-cells and functions to modulate glucagon-like peptide 1 (GLP-1) and insulin secretion when binding with its ligands. It has been shown that endogenous ligands or synthetic agonists control the release of insulin in a glucose-dependent way, contrary to insulin, sulfonylureas, and other widely used drugs, having minimal risk of hypoglycemia or other side effects [3,4,5]. Along with GPR40 expressed in pancreatic β-cells and enteroendocrine L-cells, GPR120, known as free fatty acid receptor 4 (FFA4), is also highly expressed in mature adipocytes and macrophages [6,7,8]. In addition to stimulating GLP-1 secretion from enteroendocrine cells, GPR120 expression in adipocytes and macrophages is reported to enhance glucose uptake and promote insulin sensitivity [9]. A recent study further revealed that the activation of GPR120 in mice induced brown adipose tissue (BAT) activity and promoted white fat browning [10]. Peroxisome proliferator-activated receptor (PPAR)γ agonists, especially the representative compounds, thiazolidinediones (TZDs), alleviate hyperglycemia by effectively increasing glucose uptake and insulin sensitivity and improving insulin resistance [11,12,13]. However, strong PPARγ agonists such as TZDs have limited clinical use due to side effects such as weight gain [14], edema [15], bone damage [16], and heart failure [17,18,19]. Recently, Paschoal et al. suggested that the combined therapy of low-dose rosiglitazone with GPR120 agonist compound A might improve safety by reducing side effects and increasing the metabolic benefit on glucose uptake and insulin sensitivity [20].

In drug discovery, natural products are abundant sources of chemical entities with structural diversity. Natural products have been used to treat diabetes for decades [21]. Thus, the aim of this study was to identify a GPR40 and GPR120 dual agonist from our in-house natural compound library with little to no activation of PPARγ.

Herein, a ligand-based virtual screening approach followed by docking screening was used to select potential GPR40 and GPR120 dual agonists (Figure S1, Supporting Information). We identified crocetin as a dual agonist of GPR40 and GPR120. Crocetin, a 20-carbon dicarboxylic acid, is a natural carotenoid and commercially available constituent of *Crocus sativus* Linne (saffron) [22]. 

In recent years, studies have demonstrated that saffron (*Crocus sativus* L.) is a natural product with promising glycemic control effects. Saffron and its components have been demonstrated to have hypolipidemic, antidiabetic, and antihypertensive properties in in vitro, in vivo, and clinical trials investigations [23]. The major saffron compounds are crocin, crocetin, picrocrocin, and safranal. Among them, the carotenoid components in saffron, crocin and crocetin, have been extensively researched for their potential to decrease cholesterol and prevent diabetes. Crocins are a unique class of remarkably hydrophilic apocarotenoids, comprising the primary components of saffron. Within saffron, crocetin, the aglycone derived from crocins, naturally occurs and is biosynthetically produced through the enzymatic cleavage of crocins within biological systems [24]. Several studies have shown that crocetin possesses significant pharmacological effects in various diseases, including neuroprotective, cardioprotective, hepatoprotective, antiviral, antinociceptive, antidepressant, anticancer, antidiabetic, and memory enhancing properties [22]. However, the antidiabetic activity of crocetin has not been studied as extensively as crocin. In vitro studies suggest that 10 μM of crocetin attenuates palmitate-induced insulin resistance in 3T3-L1 adipocytes via phosphorylation of insulin receptor substrate-1 (IRS-1) serine 307 [25,26]. In vivo studies reveal that 5 μM of crocetin (5 μM) treatment therapy reduced proliferative damage to diabetic endothelial progenitor cells (EPCs) and increased caspase-3 activity, LDH release, and cell death [27]. Crocetin decreased the expression levels of IL-6, TNF-α, and IL-1β, increased the levels of antioxidant enzymes such as SOD, GSH-Px, GSH, and CAT, and increased body weight in rats with STZ-induced gestational diabetes mellitus (GDM). It also suppressed the levels of intercellular adhesion molecule-1 (ICAM-1), COX-2, and PGE2 [28]. Clinical trials involving patients who consumed saffron capsules have demonstrated significant alleviation of sleep problems, anxiety, and mild-to-moderate comorbid depression-anxiety (CDA). Additionally, other studies have reported that daily saffron consumption can enhance sleep quality in diabetic patients and exert a positive impact on their anxiety levels [22]. However, the effects of crocetin on glucose uptake and insulin and GLP-1 secretion, as well as the antidiabetic mechanism of crocetin, remain unclear.

In this study, we have successfully identified saffron’s antidiabetic component at the molecular level, highlighting crocetin as a GPR40/120 agonist. This pioneering discovery positions crocetin as a promising therapeutic candidate for type 2 diabetes, marking the first unveiling of such potential. Furthermore, we characterized crocetin’s mechanism of action and assessed its antidiabetic properties in various cell types, including mouse pancreatic beta cells (MIN6), mouse intestinal neuroendocrine cells (STC-1), and embryonic mouse fibroblast (3T3-L1) adipocytes, shedding light on its potential role in diabetes management. 

## 2. Materials and Methods

### 2.1. Cell Culture and Differentiation

Mouse 3T3-L1 pre-adipocytes (Zen-Bio, Inc., Durham, NC, USA) were cultured in high glucose Dulbecco’s modified Eagle’s medium (DMEM; LM 001-07, Welgene Biotech Co., Ltd., Gyeongsan-si, Republic of Korea) supplemented with 10% bovine calf serum (BCS; Thermo Fisher Scientific Korea Ltd., Seoul, Republic of Korea) and 1% 100× antibiotic–antimycotic solution (Welgene Biotech Co., Ltd., Gyeongsan-si, Republic of Korea) at 37 °C in 5% CO_2_. Cell differentiation was initiated by adding DMEM supplemented with 10% fetal bovine serum (FBS; Welgene Biotech Co., Ltd., Gyeongsan-si, Republic of Korea), 1% 100× antibiotic–antimycotic solution, 0.5 mM isobutylmethylxanthine (IBMX; Merk KGaA, Darmstadt, Germany), 1 µM dexamethasone (Sigma-Aldrich, Saint Louis, MI, USA), and 5 µg/mL insulin (Merck KgaA, Darmstadt, Germany) when 3T3-L1 cells reached 100% confluence. Subsequently, the cells were incubated for 48 h at 37 °C in 5% CO_2_. Then, the culture medium was changed to DMEM supplemented with 10% FBS, 1% 100× antibiotic–antimycotic solution, and 5 μg/mL insulin (differentiation medium II). After an additional 48 h of incubation, the cells were cultured in DMEM containing only 10% FBS and 1% 100× antibiotic–antimycotic solution, with medium changes every 48 h. Cells were maintained at 37 °C in 5% CO_2_. Chinese hamster ovary (CHO)-K1 cells (#CCL-61, ATCC, Manassas, VA, USA) were maintained in DMEM containing 10% FBS and 1% 100× antibiotic–antimycotic solution at 37 °C in 5% CO_2_. The MIN6 cells (#C0018008, AddexBio Korea, Seoul, Republic of Korea) were cultured in DMEM containing 15% heat-inactivated FBS (10082147, Gibco, Grand Island, NY, USA), 1% 100× antibiotic–antimycotic solution, and 55 μM β-mercaptoethanol (21985023, Gibco, Grand Island, NY, USA) at 37 °C in 5% CO_2_. The STC-1 cells (#CRL-3254, ATCC, Manassas, VA, USA) were cultured in DMEM containing 10% heat-inactivated FBS and 1% 100× antibiotic–antimycotic solution. All of the reagents are reported as their final concentrations in solution.

### 2.2. SRE and CRE Reporter Luciferase Assay

The CHO cells were plated in 96-well white plates (Corning^®^ Costar^®^, Sigma-Aldrich, Saint Louis, MI, USA) at a concentration of 1.5 × 10^4^ cells per well in DMEM containing 10% FBS and 1% 100× antibiotic–antimycotic solution and incubated for 24 h at 37 °C in a 5% CO_2_ environment. The SRE (pGL4.33[luc2P/SRE/Hygro]) and CRE (pGL4.33[luc2P/CRE/Hygro]) plasmids were acquired from Promega (Madison, WI, USA) and cotransfected into CHO cells with GPR40 and GPR120 using the lipofetamine™ 3000 (L3000015, ThermoFisher, Waltham, MA, USA) as the transfection reagent. Following a 3 h incubation, the transfected cells were treated with the compounds of interest, while control cells were treated with 0.1% DMSO in culture medium and incubated for an additional 24 h. Luciferase activity was quantified using the Bright-Glo™ Assay System (Cat. #E2620; Promega, Madison, WI, USA). All individual in vitro assays for determining the average efficiencies were repeated more than three times with triplicate wells for each treatment.

### 2.3. PPARg Transactivation Activity

Briefly, CHO cells were plated in 96-well plates at a density of 1.5 × 10^4^ cells per well in DMEM containing 10% FBS and 1% 100× antibiotic–antimycotic solution. After a 24 h incubation, the cells were cotransfected with pPPRE-TK-Luc (addgene, #1015) and pCMV6-hPPARG-GFP (RG201538, OriGene Technologies, Inc., Rockville, MD, US) using Lipofetamine 3000 (Cat. # L3000015, Invitrogen, Waltham, MA, US). Following a 3 h transfection, the cells were treated with the compounds of interest and incubated for an additional 24 h. Control cells were treated with 0.2% DMSO in culture medium. Luciferase activity was assessed using the Bright-Glo™ Assay System (Cat. #E2620; Promega, Madison, WI, USA). Each individual in vitro assay for determining the average efficiencies was performed three times with triplicate wells for each treatment.

### 2.4. Insulin Secretion Assay

Briefly, MIN6 cells were seeded in 96-well plates (5 × 10^5^ cells/well) and incubated overnight to achieve 90% confluence. 1M glucose stock was used to prepare fresh 3 mM glucose KRBB buffer or 17 mM glucose KRBB buffer. Then, the culture medium was aspirated, and the cells were washed twice with 4-(2-hydroxyethyl)-1-piperazineethanesulfonic acid (HEPES)-balanced Krebs/Ringer bicarbonate buffer (KRBB) containing 0.5% bovine serum albumin (BSA, 9048-46-8, Sigma-Aldrich, St. Louis, MI, USA). Then, the cells were starved at 37 °C for 2 h in 3 mM glucose KRBB buffer. After starvation, the culture medium was changed via the indicated compounds at 37 °C for 2 h in KRBB buffer containing 3 mM or 17 mM glucose. The supernatant was collected after centrifugation and stored at −80 °C until insulin assay. Insulin levels were determined using a mouse high-range insulin enzyme-linked immunosorbent assay (ELISA) kit (80-INSMSH-E01, ALPCO, Windham, NH, USA). All individual in vitro assays for determining the average efficiencies were performed more than three times using triplicate wells for each treatment.

### 2.5. GLP-1 Secretion Assay

In brief, STC-1 cells were plated in 96-well poly-D-lysine coated plates (354640, Corning^®^, New York, NY, USA) at a concentration of 1 × 10^5^ cells per well in the culture medium. The plate was subsequently incubated for 48 h at 37 °C in a humidified 5% CO_2_. Following incubation, cells were gently washed with 200 μL prewarmed Hank’s balanced salt buffer (HBSS, SH30268.02, Hyclone, Logan, UT, USA) with 0.1% BSA to remove background secretions. Subsequently, the cells were subjected to a 3 h period of serum starvation in HBSS with 0.1% BSA. The treatment compounds were diluted in DMEM with 0.1% BSA supplemented with 5 μM the dipeptidyl peptidase 4 (DPP-4) inhibitor, KR-62436 (761414-79-3, Sigma-Aldrich), and incubated for 1 h at 37 °C. The cell supernatant was collected following centrifugation and stored at −80 °C until analysis. The levels of secreted GLP-1 were quantified using the mouse/human/rat GLP-1/glucagon-like peptide 1 ELISA kit (LS-F412, LSBIO, Seattle, WA, USA).

### 2.6. Glucose Uptake Assay

Mature 3T3-L1 cells were initially subjected to a 16 h period of serum starvation in low-glucose DMEM (2323667, Gibco BRL, Thermo Fisher Scientific Korea Ltd.). Subsequently, they were either treated for an additional 2 h with crocetin or 30 min with 100 nM insulin in glucose-depleted DMEM (11966025Gibco BRL, Thermo Fisher Scientific Korea Ltd.). Following treatment, the cells were exposed to 100 µM 2-NBDG (Thermo Fisher Scientific Korea Ltd., Seoul, Republic of Korea) at 37 °C for 1 h. After incubation, the cells were gently washed with precooled PBS, and the fluorescence intensity was quantified (Ex/Em = 485/535 nm) using a fluorescence microplate reader (VictorTM X4, PerkinElmer, Waltham, MA, USA).

### 2.7. Western Blotting

The 3T3-L1 preadipocytes were seeded in six-well plates at a concentration of 1.5 × 10^5^ cells/well in a final volume of 2 mL and differentiated as previously described. Then, the cells were incubated in low-glucose DMEM. After overnight incubation, the medium was changed to glucose-depleted DMEM. The cells were then treated with different concentrations of the compound in the absence or presence of 100 nM insulin and incubated for 1 h. The cells were harvested by RIPA buffer (Sigma-Aldrich, Saint Louis, MI, USA) containing a protease inhibitor cocktail (Roche Korea, Seoul, Republic of Korea), and proteins (20 µg) from each lysate were separated by 10% sodium dodecyl-sulfate polyacrylamide gel electrophoresis (SDS-PAGE) and transferred onto polyvinylidene difluoride (PVDF) membranes (Merk KgaA, Darmstadt, Germany), maintained at 200 mA for 120 min. The membranes were then blocked with 5% skim milk in 0.1% tris-buffered saline with 0.1% Tween^®^ 20 Detergent (TBST) for 1 h at room temperature. The membranes were then probed overnight at 4 °C with primary antibodies in 0.1% BSA. Secondary antibodies were used at a concentration of 1:5000 for 1 h at room temperature. Immunoreactive bands were marked using EzWestLumi plus detection reagents (ATTO Corporation, Tokyo, Japan) and detected by a LuminoGraph II imaging system (ATTO Corporation, Tokyo, Japan). The antibodies used were anti-p-Akt (cat.# 4060, Cell Signaling Technology, Inc., Beverly, MA, USA), anti-t-Akt (cat.# 4691, Cell Signaling Technology, Inc., Beverly, MA, USA), and anti-beta-actin antibodies (cat.# GTX109639, GeneTex, Irvine, CA, USA).

### 2.8. Structure-Based In-House Library Search

From the in-house library composed with 1158 natural compounds, we conducted a similarity search on our hit compounds with the Canvas similarity and Clustering module in Schrödinger. Tanimoto similarity metric was used, and candidates were selected via similarity score and visual inspection.

### 2.9. Molecular Modeling

Ligand docking was carried out using Schrödinger Maestro 2020-4 in Windows 10 system (Schrödinger Release 2020-4: Schrödinger, LLC, New York, NY, USA, 2020). Liga 19nds were prepared by using the 2D sketch module and Ligprep module. To facilitate docking, all possible ionization states of the ligands, including the neutral state, were generated under physiological pH conditions. The X-ray and Cryo-EM structures of human GPR40 (PDB ID: 5TZR and 5TZY) and GPR120 (PDB ID: 8ID4 and 8ID8) were obtained from the RCSB Protein Data Bank (https://www.rcsb.org/ (accessed on 19 May 2023)) [29] and were prepared using the protein preparation module. Prime was employed to replace any missing side chains, while extraneous water molecules were eliminated. For XP-docking, the ligand docking module employed XP-glide with default settings, and no constraints were applied. The co-crystallized ligand was used to define the centers of the grid boxes, with all grid boxes set to 25 Å. A total of 20 poses were generated for each ligand and exported for manual comparison.

### 2.10. Chemicals

The 1158 natural compounds were obtained from Biopurify Phytochemicals Ltd., Chengdu, China, without further purification.

### 2.11. Statistical Analysis

GraphPad Prism 7 (GraphPad Software, Inc., San Diego, CA, USA) was used to analyze the data and find significant differences. Single comparisons between the two experimental groups were performed using an unpaired Student’s *t*-test. Multiple comparisons were performed using a one-way ANOVA followed by a Tukey’s test. Data are presented as mean ± standard deviation (SD).

## 3. Results

### 3.1. Identification of Crocetin as GPR40/120 Dual Agonist

The screening strategy and diverse criteria were combined to choose hit compounds, as shown in Supporting information, Figure S1. Firstly, to save time and cost, 48 compounds representing different skeletons were selected from our in-house natural product library (Table S1) and their activity were evaluated by reporter assay. The reference compounds were AMG1637, a full GPR40 agonist, and GSK13764, a GPR120 agonist. Among the selected 43 natural compounds (Supporting information, Table S1), auraptene (a monoterpene coumarin compound) and ricinoleic acid (an unsaturated omega-9 fatty acid) exhibited the strongest activation of GPR40 and GPR120. 

To find more potential agonists, we next performed a similarity-search screening using auraptene and ricinoleic acid as templates from a commercial natural compound library comprised of 1158 compounds. A combined total of 46 compounds with a similarity score greater than 0.11 were screened for docking (Table S2). The final binding poses were ranked according to their expected binding affinity generated by the scoring function implemented in the Schrödinger program. The top 20 compounds were selected after visual inspection and their activities were assessed using the reporter assay. Several compounds demonstrated modest activities against GPR40 and GPR120. Among them, crocetin was identified as the most active compound since it exhibited greater than 90% activation of GPR40 and GPR120 at a concentration of 20 μM (Supporting information, Table S2). Thus, we selected crocetin as our lead compound for further mechanism evaluation. To our surprise, even crocetin exhibited 101.56% and 114.63% of Emax toward GPR40 and GPR120, respectively. In contrast, the Emax of crocin was only 6.00% and 5.35% for GPR40 and GPR120, respectively (Supporting information, Table S1). As a result, although crocin, a glycoside of crocetin, is the main component of saffron’s antidiabetic effect, we discovered that crocetin might also be a promising agent as a GPR40 and GPR120 dual agonist. This compound has a polyunsaturated conjugated acid structure containing four methyl groups on the side chains and seven conjugated double bonds, which include a *trans*-form and *cis*-form. In general, the *trans*-form is more stable than the *cis*-form [30]; therefore, this study mainly discusses the bioactivity of the *trans*-form.

**Figure 1:**
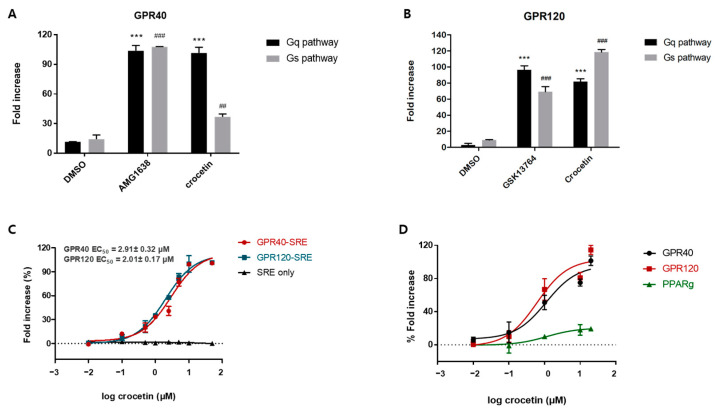
G-protein-coupled receptors (GPR)40 and GPR120, and peroxisome proliferator-activated receptor (PPAR)γ transcription activity of crocetin. GPR40 (**A**) or GPR120 (**B**) transactivation by crocetin and positive control. The transcriptional activity was determined in Chinese hamster ovary (CHO) cells transiently cotransfected with GPR40 or GPR120 plasmid and SRE-Luc (Gq) or CRE-Luc (Gs). After 3 h of transfection, cells were treated with positive control (10 μM of AMG1638 or 10 μM of GSK13764) and compounds (10 μM) for an additional 24 h. ## *p* ≤ 0.005; ### *p* ≤ 0.001; *** *p* ≤ 0.001 vs. dimethylsulfoxide (DMSO) alone by *t*-test. (**C**) Concentration-dependent SRE-Luc reporter assay in the absence or presence of target receptor plasmid. (**D**) Target selectivity test of crocetin. Data are presented as mean ± standard deviation (*n* = 3). Dashed line: Baseline at 0%.

Based on recent studies, it is believed that GPR40 and GPR120 coupled with either Gq or Gs proteins depending on the different types of agonists. It was found that the secretion of incretin is mainly dependent on the activation of the Gq protein, while further activating the Gs protein produces an additional incretin response [31]. Therefore, we determined the signaling pathway mediating the effects of crocetin. As shown in Figure 1A,B, crocetin demonstrated 9.18- and 42.45-fold higher values than dimethylsulfoxide (DMSO) in the serum response element (SRE)-Luciferase (Luc) (Gq) reporter assay, with EC_50_ values of 2.91 and 2.11 μM (Figure 1C), respectively. In contrast, crocetin only showed 2.60- and 1.57-fold higher values than DMSO in the cAMP response element (CRE)-Luc (Gs) assay. These data revealed that crocetin can strongly activate the Gq pathway and moderately activate the Gs pathway, demonstrating a dual mode of action which induced the release of insulin.

Fatty acids can also activate PPARγ, an antidiabetic target, with limited clinical utility due to undesirable side effects. Therefore, in order to verify the selectivity for GPR40 and GPR120, we ascertained the PPARγ transactivation activities of crocetin. As shown in Figure 1D, even at the concentration of 20 μM, crocetin still demonstrated weak selectivity for PPARγ, lowering the possibility of unforeseen side effects significantly.

### 3.2. Crocetin Is an Orthosteric Full Agonist of GPR40 and GPR120

The binding site of crocetin was investigated. The orthosteric site is the binding pocket for endogenous ligands of GPCRs, while the binding of other chemicals with receptors in a noncovalent manner (e.g., activating the receptor or blocking the effects of an endogenous agonist) does not inherently have to occur in the same region. Efficacy and affinity are the two key properties that determine how the ligands act on GPCRs [32]. 

**Figure 2:**
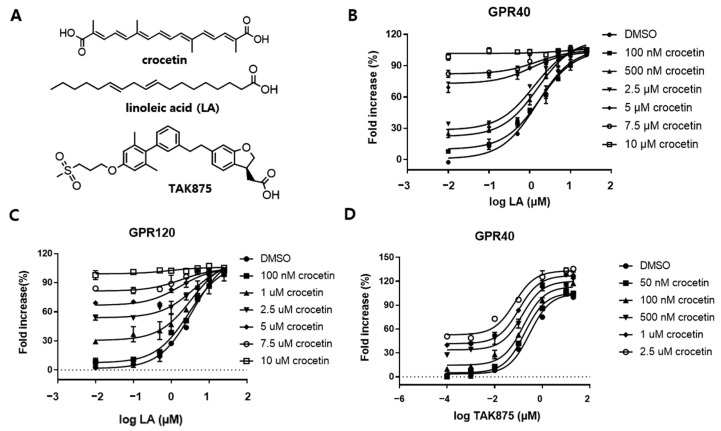
Functional assay evaluation of crocetin. (**A**) Chemical structures of the three compounds used to predict binding sites. (**B**,**C**) Functional experiments of increasing crocetin concentrations on dose−response curves of LA. (**D**) Crocetin shows synergism with the partial agonist TAK875 on GPR40. Data are presented as mean ± standard deviation (*n* = 3). Dashed line: Baseline at 0%.

Free fatty acids (FFA) are present in circulation and bind to both GPR40 and GPR120 endogenously; thus, the binding site of FFAs is identified as the orthosteric binding pocket. To explore the synergistic effect of crocetin and FFAs, we performed the SRE reporter assay. Linoleic acid (LA), a potent and effective long-chain FA, was used to investigate the binding activation site of crocetin on GPR40 or GPR120. As shown in Figure 2B,C, even though crocetin showed significant agonism toward both GPR40 and GPR120, when we fixed the concentration of crocetin with values equal to LA and coapplied it with LA, no shift was observed in the maximal response and potency. The functional assay response curve fits well in the pharmacological characterization curve of orthosteric ligands [32]. This result suggests that crocetin might be a full agonist and binds to the orthosteric binding pocket of GPR40 and GPR120. 

Furthermore, based on the crystal structure of GPR40 (PDB ID: 5TZY), the full agonist binding site could also serve as an FFA binding site and exhibit positive cooperation between partial agonists such as TAK875 [33]. Thus, we further investigated the cooperative effect between crocetin and TAK875. As shown in Figure 2D, crocetin demonstrated a significant shift toward the left on the concentration−response curve, indicating that the binding of crocetin might improve the activity of GPR40 partial agonists. 

Collectively, these data suggest that crocetin is a full orthosteric agonist of GPR40 and GPR120 and a positive modulator of TAK875 on GPR40.

### 3.3. Crocetin Stimulates Insulin and GLP-1 Secretion

**Figure 3:**
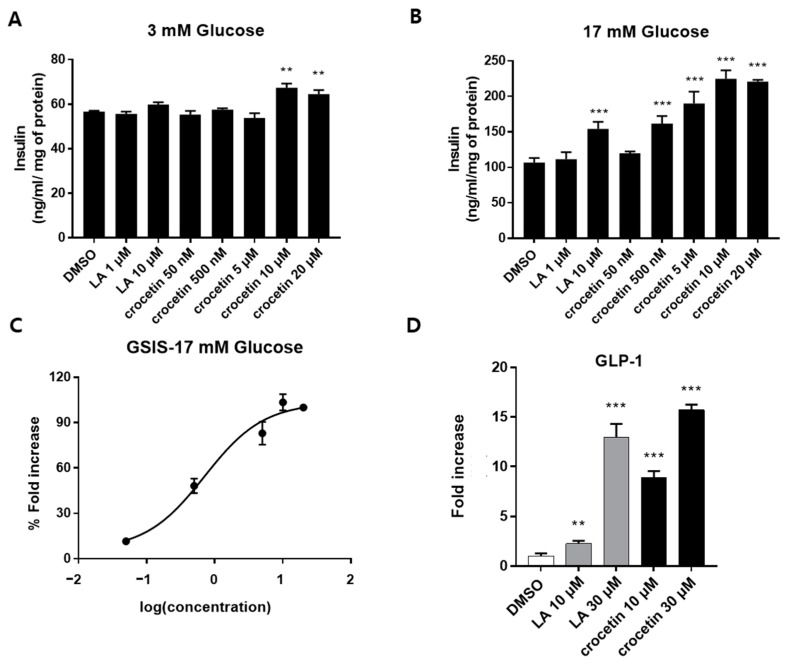
(**A**,**B**) Evaluation of GSIS by crocetin in MIN6 cells, in comparison with linoleic acid (LA). (**C**) Concentration-dependent insulin secretion curve of crocetin. (**D**) Evaluation of GLP-1 secretion by crocetin in STC-1 cells. The data are presented as mean ± standard deviation (*n* = 3). ** *p* ≤ 0.01, and *** *p* ≤ 0.001 vs. dimethylsulfoxide (DMSO) alone by using a one-way ANOVA for multiple comparisons, followed by Tukey’s test. Data are presented as mean ± standard deviation (*n* = 3).

To assess crocetin-induced insulin secretion, we conducted glucose-stimulated insulin secretion (GSIS) assays. The common in vitro study of GSIS suggested that pancreatic beta cells could be best characterized by a dynamic stimulation index obtained using a glucose step between 3–5 and 14–17 mM [31]. Seeing that the activation of GPR40/120 stimulates insulin secretion in a glucose concentration-dependent manner, insulin secretion in MIN6 cells was measured at 3 mM and 17 mM glucose. Linoleic acid was employed as a positive control to prove the functionality of the GSIS assay system. As illustrated in Figure 3A, when MIN6 cells are exposed to 3 mM glucose, high crocetin concentrations (10 μM and 20 μM) cause the cells to release very little insulin. However, as shown in Figure 3B, crocetin significantly enhanced GSIS in the presence of 17 mM glucose compared to that induced by 10 μM of LA, showing a 0.71 μM EC_50_ for insulin secretion (Figure 3C). 

Intestinal L-cells emit GLP-1, an incretin hormone, which stimulates insulin secretion and exhibits a potent effect on β-cell growth and differentiation [34]. Therefore, GLP-1-based therapies also have the benefit of improving glycemic control with little risk of hypoglycemia, avoiding weight gain, reducing blood pressure, and improving β-cell function [35]. Thus, we further investigated the effect of crocetin on GLP-1 secretion in STC-1 cells. We observed that GLP-1 secretion was concentration-dependent, demonstrating a significant (*p* ≤ 0.001) increase with crocetin concentration (Figure 3D). Although the effect of crocetin in stimulating GLP-1 secretion at high concentrations (30 μM) was similar to that of linoleic acid, at low concentrations (10 μM), crocetin still strongly stimulated GLP-1 release, exhibiting potency. Consequently, crocetin appears to have a dual mechanism of action in glucose-lowering activity.

### 3.4. Crocetin Increases Glucose Uptake in Mature 3T3-L1 Adipocytes and Insulin-Independent Akt Pathway and Increases Insulin Sensitivity

**Figure 4:**
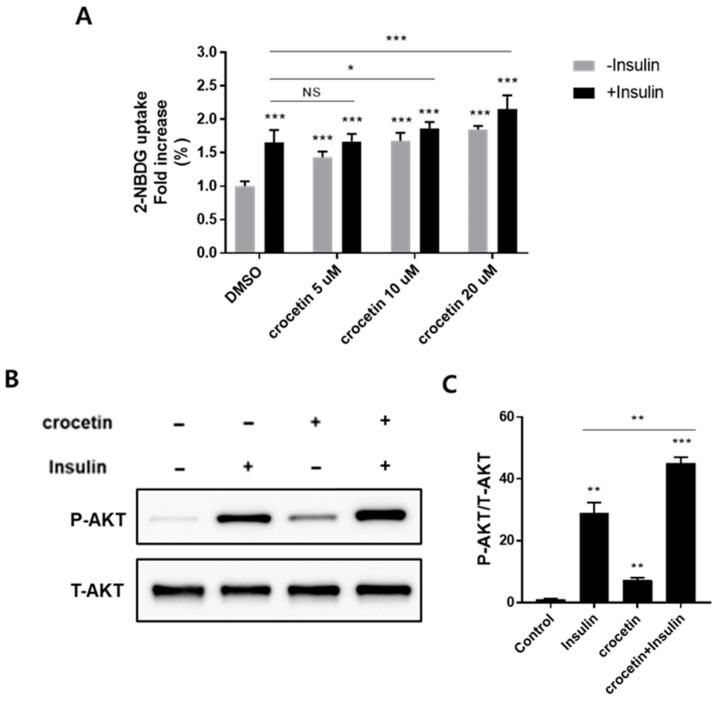
Crocetin improves glucose uptake and upregulates phosphorylated-Akt levels in differentiated 3T3-L1 adipocytes. (**A**) 2-NBDG uptake in 3T3-L1 adipocytes was measured in the presence or absence of insulin for 30 min after the cells were pretreated with crocetin for 2 h using a plate reader. Data are presented as mean ±standard deviation (*n* = 6). * *p* ≤ 0.05, ** *p* ≤ 0.01, and *** *p* ≤ 0.001 compared with the control via a one-way ANOVA for multiple comparisons, followed by Tukey’s test. NS: no significance. (**B**,**C**) Immunoblot analysis of phosphorylated-Akt levels in response to crocetin at 10 μM in the presence or absence of 100 nM insulin. The ATTO CS Analyzer 4 was used to quantify the corresponding band densities, which were then normalized to T-Akt’s (**C**). Data are presented as mean ± standard deviation (*n* = 3). ** *p* ≤ 0.01, and *** *p* ≤ 0.001 compared with the control via *t*-test.

As previously mentioned, GPR120 is highly expressed in mature adipocytes and increases glucose uptake. Thus, to clarify the antidiabetic effect of crocetin, a 2-[N-(7-nitrobenz-2-oxa-1,3-diazol-4-yl)amino]-2-deoxyglucose (2-NBDG) uptake assay was carried out using differentiated 3T3-L1 adipocytes. The 2-NBDG served as a fluorescent glucose probe. Following 3 h of starvation, differentiated 3T3-L1 adipocytes were pretreated with the indicated concentration of crocetin for 2 h, with additional incubation in the presence or absence of 100 nM insulin for 30 min. The 2-NBDG was finally added to the cells for 30 min and rinsed with cold PBS before the fluorescence intensity (Ex/Em = 485/535 nm) was measured. As shown in Figure 4A, crocetin significantly increased both basal and insulin-stimulated glucose uptake in a concentration-dependent manner. Compared with controls, with 5 μM, 10 μM, and 20 μM crocetin, glucose uptake significantly improved by 1.43-, 1.68-, and 1.85-fold, respectively, in the absence of insulin treatment. Moreover, crocetin at concentrations of 10 μM and 20 μM significantly improved insulin-stimulated uptake of 2-NBDG by approximately 1.12- (*p* < 0.05) and 1.31- (*p* < 0.001) fold, respectively, compared with the insulin-only group.

To further elucidate the mechanism of action of crocetin, we assessed the phosphorylation of Akt in mature 3T3-L1 adipocytes via Western blotting. As shown in Figure 4B and 4C, in the absence of insulin, crocetin augmented the expression level of phospho-Akt by approximately 6.0-fold, suggesting crocetin upregulated glucose uptake through the phosphorylation of the Akt pathway in an insulin-independent manner. While in the presence of 100 nM insulin, crocetin showed a synergistic Akt phosphorylation-improving effect, suggesting that crocetin might be an insulin-sensitizing agent to treat type 2 diabetes. These results indicate that crocetin treatment improves glucose uptake via activation of the Akt signaling pathway. Furthermore, crocetin exhibited no lipid accumulation effect in 3T3-L1 cells, posing little risk of fat accumulation and other side effects (Supporting information Figure S1).

### 3.5. Molecular Docking Analysis of Crocetin

**Figure 5:**
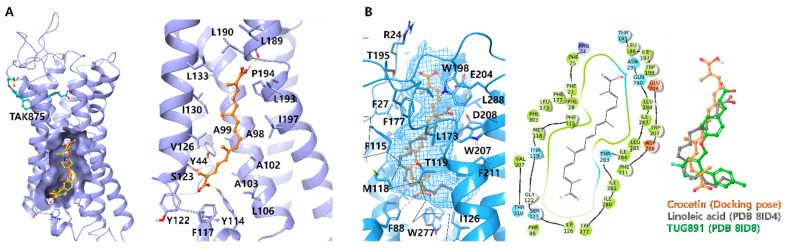
Docking model of crocetin on GPR40 and GPR120. (**A**) Superimposition of co-crystallized ligand AP8 (yellow) and docking model of crocetin (orange) and TAK875 (cyan) with GPR40 (PDB ID: 5TZY). All ligands are represented in a ball-and-stick model. Protein is represented in ribbon (faded blue) and the surface of the binding site is displayed (**left**). Docking model of crocetin on GPR40. Hydrogen bonds are represented in a yellow dashed line (**right**). (**B**) Superimposition of co-crystallized linoleic acid (gray) and docking model of crocetin (orange) with GPR120 (PDB ID: 8ID4) GPR120 and its orthogonal pocket is represented in sky blue. Transmembrane domain 3 (TM3) is simplified into a black CA trace line for better inspection. Hydrogen bonds and aromatic H-bonds are represented in yellow and purple dashed lines, respectively (**left**). 2D interaction diagram of docking model (**center**). Superimposition of structured ligand (linoleic acid-gray, TUG891-green) and docking model of crocetin (**right**).

In order to investigate the molecular underpinnings of the interactions between GPR40/120 and crocetin, computational docking studies were conducted. Orthogonal binding pockets or binding sites of full agonists are mainly discussed. For GPR40, crystal structures of GPR40-MK-8666-monoolein (PDB ID: 5TZR) and GPR40-MK-8666-AP8 (PDB ID: 5TZY) complexes were prepared for the docking of crocetin [33]. In comparison, PDB 5TZY showed an adequate binding pose and docking score because the conformation of the loop between TM3 and TM4 was secured (XP-GScore: −8.126), while it was missing in PDB 5TZR, which had a structurally similar ligand, monoolein, in the binding pocket (XP-GScore: −5.735). One carboxylate group of crocetin forms a hydrogen bond (H-bond) with Tyr114 where the carboxylate group of co-crystallized monoolein and AP8 are aligned. The other carboxylate group forms an additional H-bond with the carbonyl backbone of Leu189. A long aliphatic carbon chain interacts with the hydrophobic binding pocket of GPR40 composed of multiple lipophilic residues (Figure 5A).

Recently, the Cryo-EM structures of the GPR120–linoleic acid complex and multiple ligands have been revealed [29]. Before this structural biology study, the key residue which was considered essential for the carboxylate type of GPR120 agonists was Arg99 [36], forming multiple H-bonds at the entrance of the orthogonal pocket and the carboxylate group. However, the revealed orthogonal pocket with natural FFAs were separate from the Arg99, and the “L” configuration of the hydrophobic binding pocket recognized the double bonds of unsaturated fatty acids (Figure 5B). Crocetin tightly bonded to the orthogonal site of GPR120 and could mimic the interactions of natural ligands with multiple double bonds but could not show the “L-configuration” due to its structural rigidity (XP-GScore: −6.295). Terminal carboxylates formed aromatic H-bonds with Phe88 and potential H-bonds with Glu204 and the backbone of Phe25. The docking simulation results demonstrate that crocetin mimics natural ligands’ binding features through H-bonds, π-π, and hydrophobic interactions. While crocin, glycoside of crocetin, could not, fit into the binding pocket due to steric hindrance. Collectively, this study reveals the binding site of crocetin which is the real active component of saffron in GPR40/120, and its potential role in the antidiabetic effect.

## 4. Discussion

In recent years, in vitro and in vivo studies and clinical trials have demonstrated that saffron is a natural product with promising glycemic control effects. Multiple studies have confirmed saffron’s beneficial effects on diabetic animals’ metabolic conditions such as hyperglycemia, dyslipidemia, and insulin resistance. However, in vitro studies on the antidiabetic potential of saffron are mainly focused on its chief ingredients, crocin. In this study, we have, for the first time, identified the molecular-level target responsible for the authentic active component in saffron which imparts its antidiabetic effect. Our findings clearly establish that crocetin, as opposed to crocin, acts as an agonist for GPR40 and GPR120, positioning it as a potential therapy option for type 2 diabetes.

GPR40 and GPR120 are highly expressed in pancreatic cells and enteroendocrine cells, participate in the potentiation of insulin by free fatty acids, and, most importantly, prevent hypoglycemic complications. Several synthetic compounds and traditional natural compounds have been reported as GPR40 or GPR120 agonists in the literature. However, no GPR40/120 ligands have been approved as antidiabetic drugs. Unfortunately, trials with the GPR40 agonist TAK875 were stopped in phase III, owing to the liver toxicity associated with its chemical structure. In contrast, crocetin is a plant-derived compound from saffron, which is treated as a natural healthcare product, owing to its safety. Thus, crocetin may find application in the treatment of T2DM as a dual agonist of GPR40 and GPR120.

After conducting the ligand-based similarity search, docking, and cell-based reporter assay screening, crocetin was selected as the most active compound which exhibited an agonistic effect against GPR40 and GPR120 with an EC_50_ value of 2.91 ± 0.32 μM and 2.01 ± 0.17 μM, respectively. In the kinetic study, crocetin was identified as an orthosteric full agonist, which moderately activated both Gq and Gs pathways and revealed positive cooperation with the partial agonist TAK875 on GPR40. It was observed that both MIN6 and STC-1 cells displayed excellent insulin and GLP-1 secretion upon treatment with crocetin. This suggests that the dual agonist exhibited a powerful and dual mechanism of action for stimulating insulin secretion. Furthermore, as GLP-1 levels were also elevated in crocetin-treated enteroendocrine cells, this dual impact may arise during in vivo investigations when crocetin may directly affect insulin secretion or boost incretin secretion.

However, in comparison to adipocyte models, myocyte models have been used more extensively in vitro to study metabolic disease progression. In our case, we are working on the adipocyte models to explore the glucose uptake effect of crocetin, since GPR120 is likewise barely expressed in muscle but abundantly expressed in mature adipocytes [9]. In mature 3T3-L1 adipocytes, activation of the Akt pathway is one of the main mechanisms of glucose absorption via the activation of the signaling cascade involving PI3-kinase through the activation of GPCRs. However, unlike other insulin-dependent pathways via the insulin receptor substrate (IRS) family or tyrosine phosphatases, the PI3-kinase activation through GPCRs is dependent on Gq protein recruitment in an insulin-independent manner. Thus, in the absence of insulin, crocetin still exhibited a significant effect on Akt phosphorylation, while in the presence of insulin, crocetin showed synergistic Akt phosphorylation-improving effects, suggesting that crocetin could increase insulin sensitivity and shows potential as an insulin-sensitizing agent to treat type 2 diabetes. Nonetheless, while our research has substantiated the GPCR-dependent nature of glucose uptake, the conclusive characterization of the specific involvement of GPR40 and GPR120 in the antidiabetic function necessitates the utilization of GPR40 and GPR120 antagonists or gene silencing techniques to furnish direct evidence.

Since the primary mechanism for glucose uptake into fat cells is glucose transporter 4 (GLUT4)’s translocation to the cell surface, analysis of the GLUT4 translocation was not conducted in this study. Additional studies are required to measure the basal- and crocetin-stimulated translocation of GLUT4 to the plasma membrane in adipocytes. Nonetheless, it is reasonable to anticipate that the influence of crocetin may become more evident in subsequent in vivo analysis. This expectation is based on the established role of insulin in regulating glucose metabolism as it facilitates the translocation of intracellular GLUT4 to the cell surface. Moreover, crocetin’s capacity to stimulate insulin secretion from pancreatic cells can further enhance glucose uptake in adipocytes, potentially showcasing its effects more prominently in in vivo studies.

In conclusion, our research has unveiled a novel insight into the molecular basis of saffron’s antidiabetic effect. At the molecular level, we have pinpointed the key component and its target receptors. Notably, we have established that crocetin, distinct from crocin, acts as a dual orthosteric full agonist of GPR40 and GPR120, effectively promoting insulin and GLP-1 secretion while enhancing cellular glucose homeostasis. This discovery positions crocetin as a potent and groundbreaking candidate for diabetic therapy. To advance its development for clinical applications, further investigations involving in vivo studies to assess plasma glucose and insulin levels are imperative.

## Data Availability

All study data are provided in the manuscript and Supplementary Materials. Detailed methods and additional data are available upon request from the corresponding author.

## Supplementary Materials

The following supporting information can be downloaded at: https://www.mdpi.com/article/10.3390/nu15224774/s1, Figure S1: Schematic of the screening strategy and various criteria applied to select the lead compounds; Figure S2: Lipid accumulation effect of crocetin; Table S1: SRE-Luciferase reporter assay results of the 48 compounds; Table S2: SRE-Luciferase reporter assay results of the 22 hit compounds.
