# Supplementary material for: Identification of Crocetin as a Dual Agonist of GPR40 and GPR120 Responsible for the Antidiabetic Effect of Saffron
Source: Nutrients. 2023 Nov 13;15(22):4774. doi: 10.3390/nu15224774 (PMC10675071; doi:10.3390/nu15224774)
Supplement: Supplementary file 1 [file nutrients-15-04774-s001.zip › nutrients-2703328-supplementary.pdf]

Supporting Information

Identification of Crocetin as a Dual Agonist of GPR40 and GPR120 Responsible for the Antidiabetic Effect of Saffron

Xiaodi Zhao [1], Dohee Ahn [2], Gibeom Nam [1], Jihee Kwon [1], Songyi Song [1], Min Ji Kang [1], Hyejin Ahn [1] and Sang J. Chung [1,2,*]

[1] Department of Biopharmaceutical Convergence, Sungkyunkwan University, Suwon 16419, Republic of Korea; zhaoxiaodi1019@gmail.com (X.Z.); skarlqja12@g.skku.edu (G.N.); yg1549@naver.com (J.K.); songe1997@naver.com (S.S.); minjii_q@naver.com (M.J.K.); hyejin0930@skku.edu (H.A.)

[2] School of Pharmacy, Sungkyunkwan University, Suwon 16419, Republic of Korea; ehgml94@naver.com

* Correspondence: sjchung@skku.edu; Tel.: +82-31-290-7703

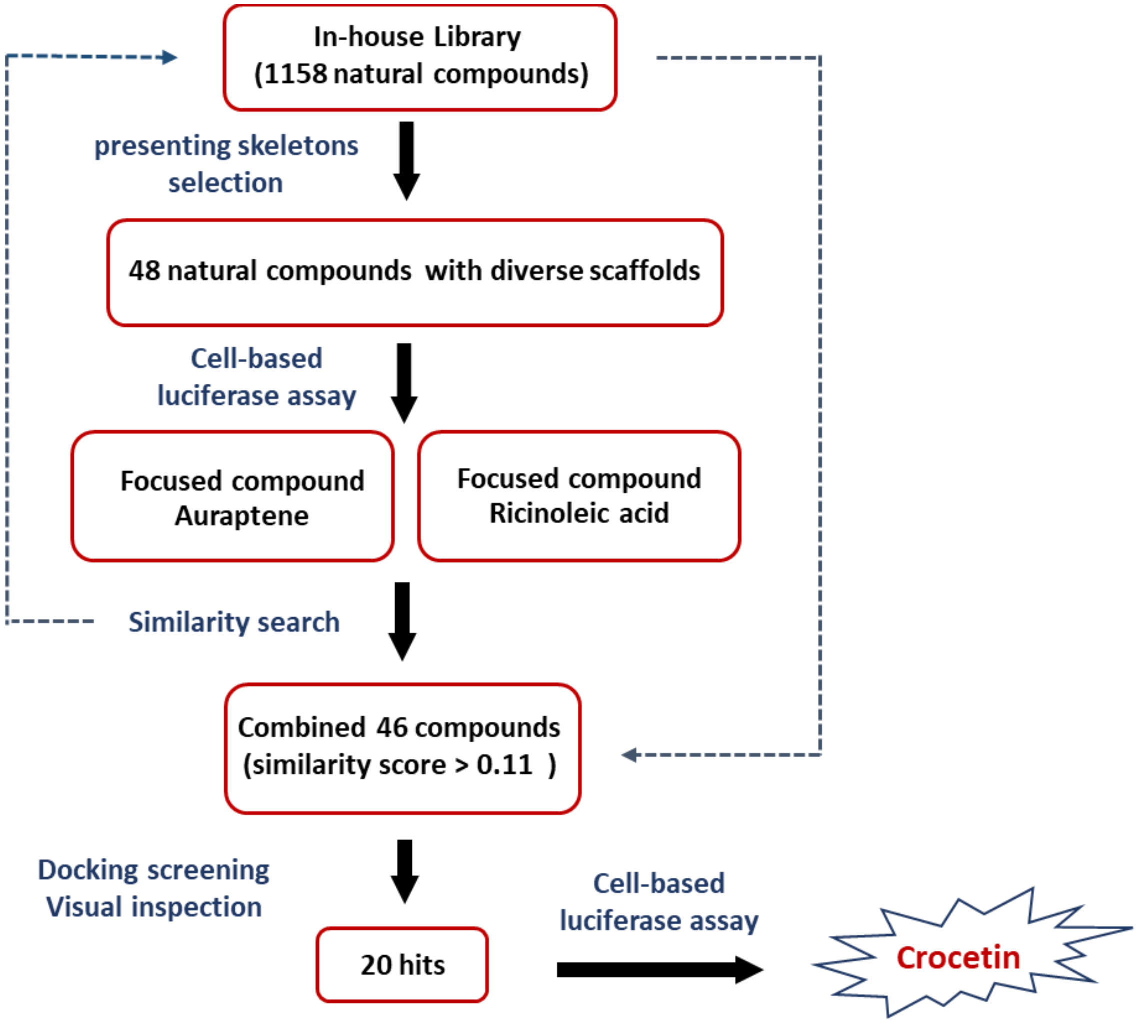

Figure S1. Schematic of the screening strategy and various criteria applied to select lead compounds.

Table S1. SRE-Luciferase reporter assay results of the 48 compounds

Compound	GPR40 Emax (%) [a]	GPR120 Emax (%) [b]
1,2,3,4,6-o-Pentagalloylglucose	0.21 ± 0.85	0.20 ± 2.84
1,5-Dicaffeoylquinic acid	5.37 ± 2.91	2.50 ± 0.99
Acetate gossypol	0.34 ± 2.12	1.55 ± 1.44
Chikusetsusaponin Iva	7.74 ± 1.16	4.03 ± 0.48
Chlorogenic acid	7.92 ± 4.70	21.18 ± 6.92
**Auraptene**	**81.36 ± 5.71**	**40.06 ± 4.97**
Chrysophanol	1.75 ± 0.87	0.43 ± 2.93
4'-Demethylepipodophyllotoxin	1.58 ± 1.50	23.91 ± 1.75
Corilagin	2.50 ± 5.25	5.27 ± 1.31
Crocin	6.00 ± 2.57	5.35 ± 0.90
Cyanidin chloride	0.20 ± 1.32	8.71 ± 2.58
desmethylbellidifolin	2.00 ± 0.61	5.47 ± 6.12
Ginsenoside Ro	2.34 ± 0.17	8.03 ± 2.38
Elllagic Acid	0.79 ± 0.25	7.19 ± 0.71
3,4-Dicaffeoylquinic acid	0.77 ± 4.17	7.60 ± 2.91
Gallic acid	1.30 ± 0.54	12.91 ± 5.76
Gambogic acid	NA	NA
**Ricinoleic acid**	**79.82 ± 6.46**	**13.54 ± 1.01**
ligustroflavone	4.77 ± 3.60	0.62 ± 6.99
Gomisin D	40.82 ± 8.41	14.92 ± 2.45
Nepetin	2.80 ± 1.64	NA
Huperzine B	12.23 ± 3.60	18.68 ± 7.58
Roburic Acid	0.44 ± 1.99	1.88 ± 0.36
Indirubin	3.16 ± 1.00	1.14 ± 0.61
Neogambogic acid	0.15 ± 0.84	NA
5,7,3',4'-Tetramethoxyflavone	4.55 ± 1.11	14.66 ± 1.69
Maslinic acid	0.53 ± 4.24	NA
Myricetrin	7.75 ± 0.72	10.05 ± 4.56
Nodakenin	2.13 ± 2.51	7.27 ± 9.60
Punicalagin	4.11 ± 0.24	10.77 ± 6.88
Punicalin	5.10 ± 1.90	12.81 ± 8.26
Quercetin 7-rhamnoside	2.90 ± 3.79	11.04 ± 0.62
Ranaconitine	1.71 ± 3.66	5.02 ± 0.24
Rhynchophylline	2.03 ± 4.68	9.37 ± 1.91
Sanguinarine	4.28 ± 0.90	4.12 ± 2.61
Scutellarein	6.61 ± 1.19	41.70 ± 6.78
Sodium Danshensu	0.76 ± 0.92	6.12 ± 0.23
Theaflavin	1.00 ± 0.54	23.43 ± 2.26
Theaflavin-3'-gallate	9.82 ± 1.97	13.39 ± 0.91
Theobromine	2.23 ± 3.19	6.37 ± 1.00
Tangeretin	5.45 ± 1.79	8.19 ± 6.24
Tormentic acid	0.450.41	NA
Wedelolactone	3.04 ± 2.37	3.61 ± 1.49

[a, b] SRE-Luciferase reporter assay. [a] Percent activity of test compounds at 20 μM compared to 10 μM AMG1638. [b] Percent activity of test compounds at 20 μM compared to 10 μM GSK13764. GPR40, G-protein-coupled receptor 40; GPR120, G-protein-coupled receptor 120; SRE, serum response element and DMSO, dimethyl sulfoxide. NA: no activity

Table S2. SRE-Luciferase reporter assay results of the 22 hit compounds

Compound	Structure	GPR40 Emax (%)[a]	GPR120 Emax (%)[b]
Ricinoleic acid	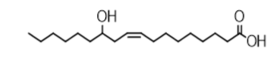	79.82±6.46	13.54±1.01
Auraptene	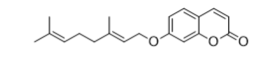	81.36±5.71	40.06±4.97
10-Gingerol	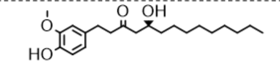	9.86±1.68	0.43±1.49
8-Gingerol	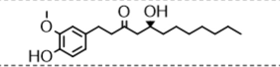	25.31±0.87	3.80±0.37
Crocetin	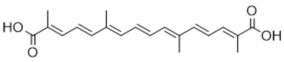	101.56±5.74	114.63±5.25
Curcumin	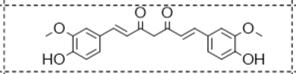	31.62±1.25	20.49±2.43
Fucoxanthin	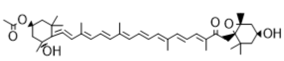	27.39±0.37	4.79±0.89
Ginkgolic Acid C13:0	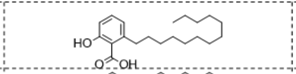	10.48±1.73	22.77±7.59
Ginkgolic Acid C15:1	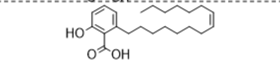	28.78±1.85	7.20±4.66
Ginkgolic Acid C17:1	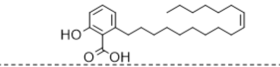	9.523±3.12	1.64±0.14
Lutein	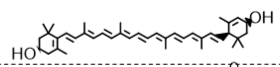	38.51±2.37	22.63±0.15
Zeaxanthin	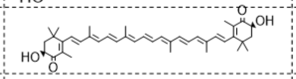	40.16±0.40	27.92±5.80
Astaxanthin	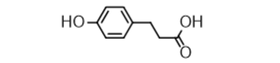	20.48±9.19	1.88±2.81
Phloretic acid	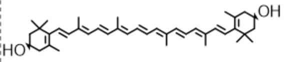	29.36±1.34	19.82±1.42
Macamide Impurity 2	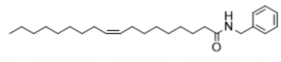	34.30±4.88	10.75±1.20
Macamide Impurity 3	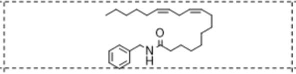	19.03±4.66	6.25±1.69
Macamide Impurity 10	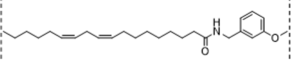	32.28±0.94	39.76±1.44
Octyl gallate	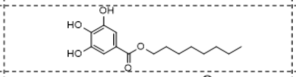	28.35±0.59	0.42±0.71
Nervonic Acid	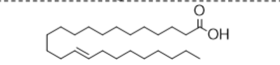	8.68±4.98	1.51±3.09
Acitretin	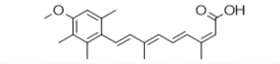	29.35±3.00	0.46±3.58
Erucic acid	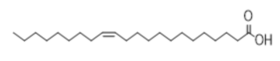	24.31±4.9	21.34±1.64
Isobavachalcone	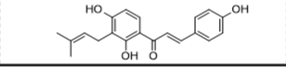	17.95±7.23	35.15±0.75

[a, b] SRE-Luciferase reporter assay. [a] Percent activity of test compounds at 20 μM compared to 10 μM AMG1638. [b] Percent activity of test compounds at 20 μM compared to 10 μM GSK13764. GPR40, G-protein-coupled receptor 40; GPR120, G-protein-coupled receptor 120; SRE, serum response element and DMSO, dimethyl sulfoxide.

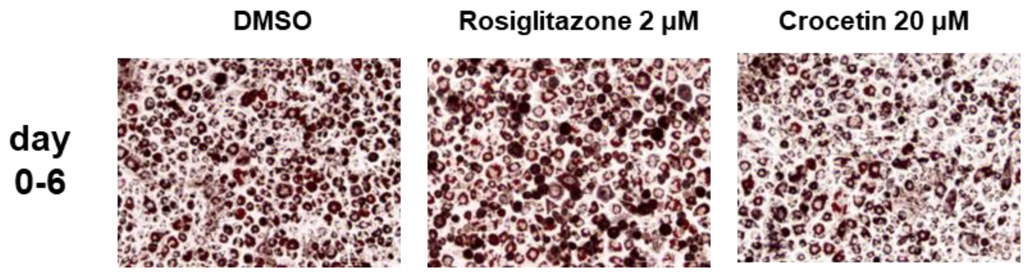

Figure S2. Lipid accumulation effect of crocetin. At the early stage of differentiation. preadipocytes of 3T3-L1 cells were differentiated by DMI-induction (DMI: dexamethasone, methylisobutylxanthine, and insulin) and treated with 20 μM crocetin or 2 μM positive control rosiglitazone simultaneously.
